# Bridging consciousness to our narrative brain: evolutionary insights

**DOI:** 10.3389/fpsyg.2025.1691355

**Published:** 2025-10-03

**Authors:** Antonio Benítez-Burraco, Francesco Ferretti

**Affiliations:** ^1^Department of Spanish, Linguistics and Theory of Literature (Linguistics), Faculty of Philology, University of Seville, Seville, Spain; ^2^Department of Philosophy, Communication and Performing Arts, Roma Tre University, Rome, Italy

**Keywords:** consciousness, language, narrative brain, storytelling, prosociality

## Introduction

There is a long tradition of attempts at bridging language and consciousness. Both are human-specific traits, even if with precursors in other species, and both are intimately intertwined. Putting it very roughly, human consciousness can be construed, to a large extent, as a sort of inner speech (Morin, 2009; Skipper, 2022). By contrast, the most developed forms of animal consciousness only entail the ability to have first-person phenomenal experiences (of the sort underwent by animals that pass the mirror test), but not a language-dependent self-awareness. One can thus expect that as language evolved more complex and versatile in our species (principally in response to environmental triggers and via a cultural process, but also as a result of brain/cognitive changes), human consciousness also became more sophisticated. Likewise, as we evolved more conscious of our external and internal world, our inner speech surely complexified to reflect the complexities of our thoughts, and if further externalized to others, this more sophisticated consciousness might have fostered the complexification of human languages. Against the background of these intricate (and hotly debated) relationships between cognition and language, and more specifically between human consciousness and inner speech, in this opinion piece, we wish to focus on the narrative dimension of our mind. We will first support the view that narratives are also tools for thinking, because they are a natural way in which we represent reality. Narratives might have thus predated language (after all, we can tell stories without a full-fledged language), but more probably, they coevolved with language (as the latter provides better ways of referring to the world and even creating new worlds). We will then reason that if human consciousness is mostly a form of inner speech, it can be said to be, to a great extent, a form of inner narrative, that is, “telling stories to oneself.” Additionally, we will show that human evolution entailed the potentiation of cognitive abilities that are key for our capacity for narrating, particularly, the ability for mental traveling (that enables us to virtually move backward and forward in time, and to places where we are not physically present) and the capacity for conceptual blending (that enables us to merge percepts and concepts belonging to different knowledge domains, and ultimately, to create fictional entities and characters). We will then show that these cognitive innovations resulted in part from changes in regions of our brain (particularly, the hippocampus, the basal ganglia, and selected cortical regions) that became modified during our recent evolution in response to changes in our socialization patterns, specifically, our increased prosociality. We will argue that these cognitive innovations might have improved our inner speech abilities (and accordingly, our consciousness), and more precisely, converted the primitive representations of the hominin narrative brain into proper (and sophisticated) narratives that enabled our ancestors to think in more complex ways, and that when shared with others, reinforced their prosocial behavior through storytelling and favored the complexification of their languages through a cultural mechanism. By all these reasons, we will conclude that, evolutionarily, language and consciousness were probably involved in a positive feedback loop, with narratives being a key component of the loop, which is something largely ignored in most evolutionary models. We will end by advancing some lines of future research aimed at delving into these issues, including the possibility of providing human-like consciousness to artificial intelligences (AIs) by improving their generative capacities for creating original narratives.

## What entails having a narrative brain

Before arguing that narratives boost (and boosted) human consciousness, we will first provide a brief definition of *narrative*, as we will be using this term here. Narratives are coherent mental representations of human experiences that result from connecting events, temporally and causally, according to the goals and reasons by one or more characters (Adornetti et al., 2022; Ferretti, 2022, 2025). Hence, *plot, time*, and *character(s)* are the essential components of a narrative, whereas *coherence* is the glue that converts such pieces into the whole we call *a narrative*. Empirical research supports the view that the components and properties of narratives are processed by different cognitive systems yet comprising a macrosystem that we will call here our “narrative brain” (see Ferretti, 2022, 2025; Ferretti and Adornetti, 2020 for details). Several components stand out as particularly relevant. First, mindreading (aka Theory of Mind) is necessary for understanding and attributing mental states to others, including the characters of a story (Ferretti and Adornetti, 2020). Second, conceptual blending (that is, our capacity to merge percepts and/or concepts belonging to different knowledge systems, in the sense of Spelke, 2000) is at the core of our ability to construct phrases and sentences, but also to metaphorize (which entails mentally connecting two entities that are not related in the real world). More specifically, conceptual blending allows us to create new words (as in compounds) or new set of words (as in phrases and sentences) that make our reference to the world more precise, but that can also refer to entities with no correlate in the real world; metaphors, on their side, allow us to depict the world in richer ways too, although they are also tools for making our languages more sophisticated through grammaticalization (see Benítez-Burraco, 2017 for discussion). Finally, two other abilities are crucially involved in the construction of narratives, and particularly in providing them with global coherence, namely, our mental time travel (MTT) ability, in turn depending on our episodic memory (EM) and our ability to project ourselves to the future; and our mental space travel (MST) ability, which enables us to project ourselves to places where we are not physically present (acting together, these two abilities are responsible for creating and processing the causal and temporal links existing between a plot's events and characters) (Ferretti et al., 2018; Race et al., 2015). EM has been further claimed to support recursion, one distinctive feature of human language that allows us to produce an unbounded set of thoughts/sentences (see Corballis, 2018 for discussion).

Studies suggest that our narrative brain can construct coherent representations of our experience in the form of narratives even in absence of language (Ferretti et al., 2018; Race et al., 2015). We will call such representations *proto-narratives*. Incidentally, this means that global coherence, as characterized above, is a property of human thought that predates language, contrary to some alternative views linking global coherence to linguistic devices (see Ferretti, 2022 for a detailed discussion). That said, language does play an active role in shaping and enriching human narratives, and this circumstance has significant implications for (the evolution of) human consciousness, as we reason below. For instance, anaphors and resumptive pronouns facilitate tracking characters through long story stretches. Likewise, a rich TAM (Tense-Aspect-Modality) helps to frame stories in accurate ways (see Benítez-Burraco, 2025b for a detailed discussion). One can safely expect that the transition from proto-narratives to fully-developed narratives was mostly a cultural process, in line with psychological models of narratives like Bruner's cultural constructivism (Bruner, 1991). One can further expect that this transition was facilitated by the emergence of full-fledged languages, which was largely the outcome of a cultural process too (Sterelny, 2016; Tamariz and Kirby, 2016; Benítez-Burraco, 2025a). As we discuss below, storytelling (that is, sharing narratives through language) seemingly played a key role in all these changes. But at the same time, these cultural changes can be expected to have remodeled our narrative brain, as new narrative practices were internalized (about this possibility, see e.g., Hutto's Narrative Practice Hypothesis, Hutto, 2009; Gallagher and Hutto, 2008). Moreover, we can also safely expect that some other brain/cognitive changes also contributed to the transition from proto-narratives to full-fledged narratives, like the potentiation of our social brain (Dunbar, 1998) or the emetgence of our language-ready brain (Boeckx and Benítez-Burraco, 2014).

## Construing human consciousness as a form of inner narrative

As noted in the Introduction, other animals seemingly have some form of consciousness, if understood in the broad sense of self-awareness. In this paper, we will distinguish between two types of consciousness: proto-narrative consciousness and narrative consciousness. Proto-narrative consciousness is the product of the cognitive processes underlying our narrative brain, as characterized in the previous section, i.e., the outcome of projective processing systems capable of providing an extended representation of experience. By contrast, narrative consciousness is the product of the same cognitive processes but if enhanced by language. For the purposes of this article, we admit that some form of proto-narrative consciousness can be found in other animals, provided the evolutionary continuity of the components of our narrative brain, particularly, the EM. EM provides humans with autonoetic self-awareness, which is “the kind of consciousness that mediates an individual's awareness of his or her existence and identity in subjective time extending from the personal past through the present to the personal future” Tulving, (1985, p. 1). According to Tulving (2002a), EM “is the only memory system that allows people to consciously re-experience past experiences Tulving, (2002a, p. 6),” thus contrasting, as also noted by Tulving (1972), with our semantic memory, which stores our knowledge of general facts without any reference to our personal experiences. Tulving (2002b) has termed “chronesthesia” this capacity of the EM to refer to a subjective time. Corballis (2015) has argued that our MTT ability presupposes chronesthesia, which is not surprising, since MTT depends on the EM. But he has further rooted our “temporal consciousness” to the hippocampus, the neural substrate of EM. Interestingly for our discussion here, in Tulving's words (2002, p. 5), “the essence of episodic memory lies in the conjunction of three concepts–self, autonoetic awareness, and subjective sensed time,” which are very close to (or core components of) human consciousness.

Clinical research supports Tulving's thesis about a fundamental distinction between EM and semantic memory, and ultimately, between two distinct ways of experiencing time: known time (i.e., being familiar with calendars, clocks, history and the like), which is associated to the latter, and lived time (i.e., having a subjective experience of time, including the capacity to recollect personal past events and to project oneself into the future), which is associated to the former (Kapur, 1999; Klein et al., 2002). Clinical cases further show that the impairment of (components of) our narrative brain (particularly, EM) co-occurs with an impairment of consciousness. Accordingly, the impairment of EM results in the loss of awareness in subjects with mild cognitive impairment (Gagliardi and Vannini, 2022). Likewise, people with autism spectrum disorders show both diminished EM and autonoetic awareness (Lind and Bowler, 2008). Interestingly too, compared to neurotypical controls, patients with disorders of consciousness exhibit reduced intersubject correlation of neural responses to narrative speech audition (Iotzov et al., 2017).

Our contention is that the cognitive systems underpinning our narrative brain can generate *per se* not only proto-narrative representations, but also certain forms of subjective experience, and ultimately, of consciousness. And since these cognitive systems, particularly the EM rooted in the hippocampus, have experienced changes in our species (see Corballis, 2019 for details), our proto-narrative consciousness resulted improved compared to other animals. At the same time, because language plays an active role in shaping and enriching human narratives, as noted, we further expect that our language abilities improved our consciousness even more. Specifically, because of the notorious external (i.e., social and cultural) nature of full-fledged narratives, our consciousness was seemingly enriched by the internalization of social and cultural phenomena, as transmitted, by storytelling. This is in fact observed during ontogeny. Early during development, the child mostly builds on her innate abilities for constructing proto-narratives, and ultimately, for developing (a basic form of) consciousness (Vandekerckhove, 2009). Later in development, as language complexifies and fully-fledged forms of storytelling are mastered, a true consciousness finally emerges (Bruner, 1987). We think that the same happened during human evolution, as we discuss in the next section.

To summarize (and to emphasize the key points of) the discussion above: humans are endowed with a proto-narrative form of consciousness which is mostly phenomenal in nature and which exhibits a notable evolutionary continuity; by contrast, we also own a narrative form of consciousness which is mostly non-phenomenal in nature and which can be regarded as a genuine human innovation. In truth, both proto-narrative consciousness and narrative consciousness are characterized by phenomenal aspects as well as narrative features, although to different degrees.

## When humans evolved more prosocial, our narrative abilities improved (and also our consciousness)

Among primates, humans stand out as featuring reduced reactive aggression together with high levels of proactive aggression (Wrangham, 2018). This results in both our noticeable prosocial behavior with relatives and familiar people, and our proclivity to engage in premeditated hostilities and escalating wars with unknown people. Such an uneven behavioral profile seemingly resulted from our recent evolutionary history, which favored cooperation (including extensive community living and co-parenting) in response to fluctuations in food availability, as climate changed during the Last Glaciation, but also to population increases during the warm periods (Hare et al., 2012; Pisor and Surbeck, 2019; Spikins et al., 2021; McCall, 2025). According to an influent view, namely the Human Self-Domestication (HSD) hypothesis, selection of less aggressive individuals triggered in our species the constellation of features that are typically observed in many domesticated animals, which subsequently facilitated the emergence of most of the human distinctive traits, notably our increased cooperation and extended social networks, and ultimately, our complex technology and sophisticated culture (see Hare, 2017; Hare and Woods, 2020 for details). Although all these innovations are hypothesized to have been fostered by the behavioral and social changes brought about by HSD, which potentiated cumulative cultural learning through increased opportunities for interacting with experts and for practicing know-hows, HSD is also thought to have produced cognitive changes in our species also contributing to the human phenotype. Importantly for our concerns here, these changes include the improvement of abilities like EM (Benítez-Burraco, 2021) and conceptual blending (Benítez-Burraco and Progovac, 2021), which, as noted, are at the core of our narrative abilities, and accordingly, of our distinctive form of consciousness. HSD is also hypothesized to have improved our language abilities very significantly (Benítez-Burraco and Progovac, 2020; Raviv and Kirby, 2023; Benítez-Burraco, 2025b). Very probably, our ancestors mostly used their increasingly sophisticated languages (and narratives) for social goals, particularly, for providing cohesion to their groups through the creation of shared stories and myths (Dunbar, 2010; Dunbar et al., 2014; Benítez-Burraco, 2025a). But because, as noted, HSD seems to have impacted as well on core components of our narrative brain and of our linguistic brain, and because HSD also facilitated the cultural complexification of human languages and of the stories we conveyed through language, we wish to argue that HSD might have potentiated our “internal speech” too, in the form, specifically, of an improved ability to tell more complex stories to ourselves. In other words, we support the view that HSD potentiated human consciousness.

## Conclusions and future lines of research

In this opinion paper, we have argued that our understanding of the nature and evolution of human consciousness can benefit from considerations about the nature and evolution of language (because human consciousness is, to a large extent, a form on internal speech) and particularly, of our narrative abilities (because human consciousness can be construed, specifically, as the ability to tell stories to ourselves about how the world is and about ways of interacting with it). Moreover, we have argued that the HSD hypothesis of human evolution is a promising evolutionary framework for clarifying the origins and properties of human consciousness.

Several lines of research can improve the approach to human consciousness we have advocated in the paper. First, looking for proxies of narrative abilities in extinct species (resembling the proxies or “windows” used in language evolution studies; see Johansson, 2005; Botha, 2016). Second, searching for precursors of our narrative brain/abilities in other animals. Third, determining patterns of impairment/preservation of our narrative brain/abilities in human-specific cognitive disorders impacting in human consciousness, such as schizophrenia. But given the objectives of this Research Topic, we propose using IAs for examining (and even modeling) the links between consciousness and language (including narratives). For instance, one could hypothesize that if Large Language Models (LLMs) are provided with episodic-like memory capacities, their narrative capacities should improve. Because LLMs are fed with huge amounts of human texts, they can be said to have ample access to the social and cultural dimensions of human narratives. Accordingly, if our hypothesis is on the right track, one prediction is that AIs will develop some form of human-like consciousness. We are not merely saying that human-like consciousness can be implemented in a machine if we provide it with access to its environment (i.e., if we grant it access to the phenomenal aspects of consciousness). Indeed, this can be safely expected. What we aim to suggest is that IAs could develop human-like consciousness even in absence of direct phenomenal experience if we provide them with a model of our narrative brain. Or more exactly, if we implement in them a simulation of the mutual dependences between the narrative components and the proto-narrative elements as found in humans, which are mediated by language and by specific cognitive systems, and which are needed to transcend the phenomenal character of conscious forms of representation of experience.
